# Associations Between Aging-Related Biomarkers, Interstitial Lung Abnormalities, and Mortality

**DOI:** 10.1093/geroni/igaa057.2698

**Published:** 2020-12-16

**Authors:** Jason Sanders

**Affiliations:** Brigham And Women’s Hospital, Wellesley, Massachusetts, United States

## Abstract

Interstitial lung abnormalities (ILA) exist in ~10% of adults >50 and associate with increased morbidity/mortality. Their pathobiology is poorly understood; age is the strongest risk factor. In the Framingham Heart Study, we determined associations between ILA and 10 blood biomarkers previously robustly associated with aging and mortality. Odds of ILA increased directly with ln-transformed GDF15 (OR [95% CI] = 3.20 [1.74-5.91], p=0.0002), TNF-αRII (2.41 [1.34-4.34], p=0.003), IL6 (1.76 [1.39-2.22], p<0.0001), insulin (1.56 [1.11-2.20], p=0.01), and CRP (1.53 [1.27-1.84], p<0.0001). Causal analysis showed GDF15 (p=0.008), TNF-αRII (p=0.004), and IL6 (p<0.0001) mediate the age effect on ILA. In adjusted survival models, only higher ln(GDF15) and ln(TNF-αRII) were associated with mortality (HR [95% CI] = 4.3 [2.3-8.1], p<0.0001 and 2.9 [1.5-5.8], p=0.002). GDF15 results were replicated in the COPDGene Study. These results suggest aging biomarkers may help risk stratify adults with ILA, and unmeasured ILA may confound prior associations between biomarkers and mortality.

